# Paediatric cancer care in a limited-resource setting: Children’s Welfare Teaching Hospital, Medical City, Baghdad

**DOI:** 10.3332/ecancer.2016.ed55

**Published:** 2016-05-24

**Authors:** Salma A Naji AL-Hadad, Mazin Faisal Farhan Al-Jadiry, Claudia Lefko

**Affiliations:** 1 Oncology Unit, Children’s Welfare Teaching Hospital, Medical City complex, Bab Almoaddam, Baghdad, Iraq; 2 Baghdad Resolve: An International Collaboration to Improve Cancer Care in Iraq; 3 40 Valley St, Northampton, MA 01060, USA

**Keywords:** children, cancer, Iraq, social determinants of health

## Abstract

There has been a lot of news coming out of Iraq in recent decades, but most of it ignores the situation for people on the ground: ordinary men, women and children who continue trying to live their lives in spite of wars, economic sanctions, violence, and social, political and cultural collapse. The challenges of maintaining and sustaining health in an environment where everything—the human spirit, education and health care systems and the health-sustaining infrastructures of housing, water purification and the electric grid—is damaged or broken are enormous.

In an August 2011 email Dr Mazin Al-Jadiry, a paediatric oncologist at Children’s Welfare Teaching Hospital in Medical City Baghdad wrote: *Really, I’m asking my/ourselves if we have the power to continue as the circumstances around us are not improving, but deteriorating on levels that we could never-have-imagined: chaos, corruption and a loss of principles which were already declining over the last 30 years. There is continuity of the obstacles. And, the number of patients is increasing. We feel we should/cannot be static, that we have to improve our abilities and expand our capacities. This puts even more of a burden on us, both physically and mentally.*

The healthcare system—along with the doctors and nurses who try to deliver healthcare—continues to struggle as instability thunders into 2016, with no end in sight. So, what are Iraqi doctors and nurses to do? And how can the global health community of doctors, nurses and institutions help in Iraq? We offer some ideas.

“...in most of the developing world, the landscape for cancer care remains bleak. There is no time to waste; we must act now. We have an epidemic on our hands. It is our moral responsibility to not only save lives, but also alleviate undue suffering.”

HRH Princess Dina Mired, Honorary co-President GTF.CCC; October 2011 Amman Jordan

Childhood cancer and leukaemia in Iraq continues to be referred to as a long-term health crisis, an oxymoron that redefines the situation from acute to ordinary. Iraqis in general are living in a chronic state of dysfunction and illness that has become normalized, “normal” in the public view, in public discourse and even, it seems, normal to the people directly affected. But the situation in Iraq is not normal.

There have been reports of a cancer crisis in Iraq for more than twenty years. Much of the evidence has been anecdotal because it has been, and continues to be nearly impossible to gather and/or process any sort of health-related data in the chaos of the last decades. There is a lack of national health data across the boards. To some extent what one can “know” about specific health problems such as cancer in Iraq comes from the records of individual clinics, hospitals and doctors. In our case, records from the paediatric oncology unit at the Children’s Welfare Teaching Hospital (CWTH) in Medical City Baghdad show patient numbers are increasing. In one four-year period the number of cancer/leukaemia in-patients doubled from 110/month in 2005 to 218/month in 2009. There were 1,330 patient admissions in 2000, increasing to 2,970 in 2014. The number of visitors to the outpatient clinic, including those with benign haematology and malignant disease, was recorded as 2,320 children in 2000, increasing to 11,432 patients in 2014. Because there is no reliable patient tracking, it is hard to know if these numbers represent newly diagnosed cancer cases or whether we are recording more patients because of the overall lack of medical facilities/cancer units. Only five new hospitals have been built in Iraq in the last thirty five years.

CWTH is a public referral and teaching hospital with 240 beds managed by the Government of Iraq. The staff are government employees. The oncology unit has a capacity of 56 beds, but the number of actual inpatients ranges from 60–90. There are an average of 300 newly diagnosed malignant cases per year, ranging from 235–398 over the last twelve years. The medical staff includes nine oncologists, one fellow, six residents (changing every two months) and twenty-five nurses. This hospital unit is one of the largest cancer facilities in the Middle East, serving almost twice as many patients as the King Hussein Cancer Hospital in neighbouring Jordan

Services are free, but somewhat limited. All childhood cancers are treated except for brain tumours as there is no space to accommodate such a programme and there is no radiotherapy centre or multidisciplinary care teaming to assist in the management of these kinds of tumours. The average registered mortality ranges from 10–20% over the last ten years. Two thirds of these deaths occurred during induction and result from the lack of optimum supportive care. About the same percentage, two thirds of patients come to CWTH in an advanced stage of disease. The median total diagnosis delay was 55 days (from 3 days to 36 months). The median physician delay was 43 days which is more than double the longest doctor delay mentioned in the literature, although the patient’s delay in seeking diagnosis/treatment is comparable to that mentioned elsewhere. This late diagnosis along with iatrogenic complications can make for a poor prognosis.

The consultant/doctor, medical fellows, and to a lesser extent, nursing staff and the junior doctors are expected to manage the child over their course of treatment at the hospital. The challenges for the medical staff are enormous. There is a chronic shortage of doctors and college-trained paediatric oncology nurses, resulting in a high patient/doctor/nurse ratio. Qualified nurses are in short supply; only 28% of the nurses on the unit at CWTH are college graduates. Most have only a secondary school education and lack specific training in nursing and medical fundamentals; they are not proficient in English, the international language of medicine.

There is no multidisciplinary team to share in the physical/medical care of the child, only doctors and the medical team—no Clinical Nurse Specialists, Occupational or Physical Therapy; no Community Nurses or hospitals, and no hospice care. There is no multidisciplinary team to meet psychosocial needs, no social workers, teachers, spiritual advisors or child life therapists who might improve the psychological state of the child or family or nurture their spiritual strength; no one who might distract them with meaningful activities.

Thus, an inadequate number of doctors are expected—indeed in order to provide adequate care, they must—do everything for the child and family, including, finding the patient a bed on the unit, supplying missing drugs, providing triage (i.e. being available for consultation by phone) and finding financial support and other resources for the family.

What should doctors do when the country where they live and work lacks the capacity to provide something as basic and important as healthcare for its population; when the hospitals lack the necessary human and financial resources to maintain their facilities or keep well-qualified medical staff? Iraqi doctors, struggling to carry on, experiencing the cumulative effects—both professional and personal—of a series of long-term crises, have found themselves in need of help. And so the team at CWTH has both reached out and been open to offers of international help and collaboration for the last two decades. The extraordinary efforts of the Iraqi medical team, combined with those of a variety of international partners have paid off in that a certain level of care has been maintained, and in some areas there have been improvements. While this is not an optimal way to provide healthcare—healthcare is the responsibility of governments—it is, nonetheless a way to carry on when there are few choices. It places a heavy burden on the shoulders of doctors at the hospital, who must not only take care of patients, but also build the institutional structure needed to provide care in the context of ongoing chaos and instability outside of the hospital.

We are in an interesting historical moment with regards to health, one which would seem to hold some promise for improving health and the healthcare system, for improving the situation for oncologists and their patients in Iraq. The legal framework that defines health as a human right is being strengthened, creating new, substantive opportunities for individuals and international human rights organizations—rightly concerned about the overall situation in Iraq after decades of economic, social and political instability—to take action on behalf of Iraqis whose overall health continues to suffer as a consequence of the ongoing chaos in the country. Two important documents, the International Covenant on Economic, Social and Cultural Rights (ICESCR) and the International Covenant on Civil and Political Rights (ICCPR) bundle six basic economic, social and cultural as well as civil and political rights into the concept known as the social determinants of health, reaffirming that “good health” depends on achieving and maintaining other rights: adequate housing, education, food, social security, decent work and “the highest attainable standard of physical and mental health” and recognising that culture and environment can be as significant as internal physical factors of disease risk [1].

In addition, the medical community is increasingly concerned about cancer and specifically the cancer divide—the gap between care in high versus in low and moderate income countries. High-profile diseases such as SARS and Ebola, and now the ZIKA virus, grab headlines and an enormous percentage of global human and financial resources, leaving cancer and other non-communicable diseases (NCDs) in the background, out of mind and out of resources. In response, in September 2011, the UN convened The High-level Meeting of the General Assembly to consider the Prevention and Control of Non-communicable Diseases. The outcome document declared non-communicable diseases as a priority for development as well as for health in LMICs [2].

Iraq is a LMIC with a high rate of paediatric cancer; it is a country that is in desperate need of development/re-development and on the wrong side of the cancer divide. The country, and in this case, the specialty medical community of paediatric oncologists at CWTH, has been in crisis and in need of assistance from the international community for many years. But what can be done in Iraq, such an unstable and dangerous country, mired for nearly four decades in national and international conflicts. It seems to us that this historical moment, with strengthened human rights language, an expanded definition of health as a human right and a focus on NCDs, offers new possibilities for global health doctors, nurses and institutions to help in Iraq.

Iraqis cannot wait for stability to return to the country. Important groundwork, a foundation for action is in place. The stage has been built, the play has been written. It’s time for the actors—International aid and global health organizations working in collaboration with Iraqis—to take their places. Let the action begin!

We would like to see the creation of international cooperative groups committed to aggregating knowledge and experiences in ways that can be effectively shared with doctors, nurses and other medical staff to improve patient outcomes in LMICs. In addition, we see a need to expand twinning projects, and other programmes that encourage and support the exchange of personnel—even, and maybe especially—in challenging sites such as Baghdad. Opportunities need to be extended to data managers, clinical pharmacists, paediatric palliative care workers and other members of multidisciplinary paediatric oncology teams. To be effective, these helping initiatives need to be designed to address the complex needs of people living and working in exhaustive and challenging settings in LMICs.

Staff and international partners are doing the best we can at CWTH, but we need much more help and support from the international community, especially, in our case, from individuals and groups focused on cancer and cancer care.

## References

[1] Partners in Health (2011). Management Guide.

[2] Knaul (2011). Global Task Force on Expanded Access to Cancer Care and Control in Developing Countries. Closing the Cancer Divide: A Blueprint to Expand Access in Low and Middle Income Countries.

